# Public Attitudes Towards COVID-19, Antibiotic Resistance, Preventive Measures: A Multi Center Cross-Sectional Study in the Arab Countries

**DOI:** 10.1007/s44197-023-00092-6

**Published:** 2023-02-23

**Authors:** Sarya Swed, Karem R. Motawea, Haidara Bohsas, Hidar Alibrahim, Amine Rakab, Wael Hafez, Nour Shaheen, Mohammad Badr Almoshantaf, Shoaib Ahmad, Sifwa Safdar, Lina Taha Khairy, Agyad Bakkour, Ali Hadi Hussein Muwaili, Dhuha Hadi Hussein Muwaili, Fatima Abubaker Abdalla Abdelmajid, Eman Mohammed sharif Ahmad, Muhammad Mainuddin Patwary, Hazem S. Ghaith, Mhd Kutaiba Albuni, Elias Battikh, Bisher Sawaf, Mohamed Elsayed, Nashaat Kamal Hamdy Elkalagi, Sheikh Shoib

**Affiliations:** 1 Faculty of Medicine, Aleppo University, Aleppo, Syria; 2 Faculty of Medicine, Alexandria University, Alexandria, Egypt; 3 Assistant Professor of Clinical Medicine, Medicine, Weill Cornell Medical College, Al Rayyan, Qatar; 4 NMC Royal Hospital, 16Th Street, Khalifa City, Abu Dhabi, UAE; 5 Medical Research Division, Department of Internal Medicine, The National Research Centre, Cairo, Egypt; 6 Ibn Al-Nafees Hospital, Damascus, Syria; 7 Allam Iqbal Medical College, Lahore, Pakistan; 8 The National Ribat University, Al-Ribat, Sudan; 9 Faculty of Medicine, Albaath University, Homs, Syria; 10 Ivano-Frankivsk National Medical University, Ivano-Frankivsk, Ukraine; 11 University of Medical Sciences and Technology, Khartoum, Sudan; 12 Nile Valley University, Atbra, Sudan; 13 Environment and Sustainability Research Initiative, Khulna, 9208 Bangladesh; 14 Environmental Science Discipline, Life Science School, Kulna University, Khulna, 9208 Bangladesh; 15 Faculty of Medicine, Al-Azhar University, Cairo, Egypt; 16 Department of Internal Medicine, Damascus University, Damascus, Syria; 17 Department of Internal Medicine, Syrian Private University, Damascus, Syria; 18 Department of Psychiatry and Psychotherapy III, University of Ulm, Leimgrubenweg 12-14, 89075 Ulm, Germany; 19 Lecturer in Internal Medicine and Tropical Medicine at Faculty of Medicine Al Arish University, Alarish, Egypt; 20 Department of Psychiatry, Jawahar Lal Nehru Memorial Hospital, Srinagar, Kashmir India

**Keywords:** COVID-19, Antibiotic resistance, SARS-COV2, Cross-sectional, Public knowledge

## Abstract

**Background and Aim:**

COVID-19 has shown how crucial awareness of the need to protect public health is to global security. Antibiotic resistance due to antibiotic misuse is seen as a worldwide health issue. Antibiotic use was significant during the COVID-19 epidemic, according to several nations. This research aims to investigate public attitudes on COVID-19, antibiotic resistance, and preventive measures during the COVID-19 pandemic in the Middle East.

**Methods:**

An online quantitative cross-sectional study in 17 Arabic nations was carried out between January 3 and March 4, 2022, using a structured questionnaire to evaluate participants’ knowledge of COVID-19, their attitudes toward the new standard during the pandemic, and their use of antibiotics, and their resistance to them. The research was available to all Arabic people over 18 nations in the middle east. A convenient snowball sampling technique was used. SPSS version 20.0 was used to analyze the data. To analyze the results, binominal logistic regression was utilized. Statistical significance was defined as a *p* value of 0.05.

**Results:**

Of the 6145 responders, 24.1% believed COVID-19 might spread to asymptomatic people, whereas 13.6% thought using antibiotics would accelerate recovery from any illness. Moreover, half of the respondents said antibiotics only work against bacteria (64.6%). 70.8% of participants adopted the necessary safety measures. More than a third of respondents strongly supported placing foreign immigrants in quarantine (33%). However, more than 50% of those surveyed (52.5%) firmly supported using face masks in all public settings. Individuals with a medical education background had 2.6 times more appropriate understanding of antibiotic resistance than others. Furthermore, participants in the 30–49 age range had a better handle on the use of antibiotics and antibiotic resistance than other respondents by 1.1 times.

**Conclusion:**

Arab Health authorities should reconsider this health issue as soon about the inadequate level of awareness toward antibiotic use, resistance, and preventative practices during COVID-19. Many suggested strategies, especially solving the irregular antibiotic prescriptions during a COVID19 pandemic, should be implemented to increase public awareness of COVID19.

## Background

COVID19 disease is caused by the SARS-COV2 virus that emerged in December 2019. It has been linked to various respiratory symptoms, ranging from mild to severe. Confounding variables such as old age, cancer, chronic disorders, and respiratory tract diseases may cause significant symptoms in some patients [1]. The most common symptoms of norovirus are mild fever, cough, fatigue, and loss of sense of smell or taste, and less common symptoms include headache, diarrhoea, and rash on the skin. The virus's incubation period extends from 5–6 days up to 14 days [1]. In response to the rapid global spread of the COVID-19 virus, the World Health Organization (WHO) has proposed a categorization for COVID-19 variants, which accounts for variations in binding proteins, clinical outcomes, antibody elimination, and potentially symptom severity and transmission. The alpha mutant increased the Risk of transmission, Intensity, and Incident mortality, whereas the beta mutant increased Infectiousness and lowered Neutralization to convalescent and post-vaccination sera. The Omicron COVID-19 mutant was discovered for the first time in South Africa in November 2021 and is considered one of the most ineffective variants of the virus. However, the omicron mutant has increased both the chance of transmission and the danger of reinfection [2]. It was recommended to apply the following supportive therapies: healthy nutrition, antipyretics, oxygen saturation, and keeping the vital signs within the normal range [3]. Many treatments were introduced, such as chloroquine, hydroxychloroquine, ivermectin, and anti-asthmatics, all of which have not been demonstrated to have a therapeutic effect against COVID-19 and may develop severe complications due to their use [4]. After the worldwide spike in COVID-19 cases, vaccination became the sole effective means of stopping the spread of the disease. In August 23, 2021, the FDA authorized the use of the Pfizer-BioNTech COVID-19 vaccine. The Pfizer vaccine was available to persons aged 16 and older; nonetheless, it is 100% effective in preventing serious illness as defined by the CDC and 95.3% effective in preventing severe disease as described by the FDA. The two-dose AstraZeneca vaccine was given to people 18 years or older, and it worked 55% of the time and stopped hospitalizations 100% of the time. While generally safe, vaccinations may cause adverse reactions such as anaphylaxis, myocarditis, and vaccine-induced immunological thrombotic thrombocytopenia [5]. However, some vaccines have been developed against the virus nowadays. The world is facing a problem: the indiscriminate use of antibiotics due to people’s lack of complete Knowledge of the mechanism of action of antibiotics and their correct effectiveness, which has exacerbated the bacterial resistance to antibiotics [6]. Because of the virus's respiratory symptoms, people thought they could get rid of it by taking antibiotics, leading to indiscriminate antibiotic use, bacteria developing resistance to drugs, or other disorders, including spinal muscular atrophy [7, 8]. Lots of treatments, such as chloroquine, hydroxychloroquine, ivermectin, and anti-asthmatics, have not been demonstrated to have a therapeutic effect against COVID-19 and may develop severe complications due to their use [4]. Antibiotic self-prescription has been more common in the Arab world, and a runny nose and sore throat are typical of this problem. The most common cause is a random prescription from a family or community pharmacy, with an economic and educational effect on antibiotic use [9, 10]. Antibiotics were self-medicated by 32–62% of the population in Jordan, 32–42% in Lebanon, 56% in the Emirates, 20% in Tunisia, 60% in Yemen, and 73.9% in Sudan. Moreover, the percentage of antibiotic self-medication among Palestinian high school and college students was 98%, 46% in Libya, 40% in the United Arab Emirates, and 48% in Saudi Arabia [11]. Knowledge gaps, conventional attitudes, and behavioral tendencies can all be revealed by KAP surveys, which can help people, comprehend and behave more effectively. Because of the limited research infrastructure and weak health systems in Arab countries, official KAP investigations are rarely done. We detected poor expertise regarding antibiotics utilization and Knowledge of the COVID-19 virus during the COVID19 outbreak. It is critical to research this area throughout this period to measure the genuine level of awareness among the Arabic community and compare it to that of other populations.

After completing an extensive study on the behavior of the residents of the Arab world, we organized this study to ascertain COVID-19 knowledge, attitude, and practices regarding COVID-19 and antibiotics’ use in the Arab countries.

## Methods

### Study Design and Setting

We conducted cross-sectional research to gather data from the Arabic world using an online questionnaire based on an initial questionnaire for a study conducted in Malaysia [9], and translated into Arabic to be appropriate for the Arab world.

The inclusion criterion included Arabic people above 18 who lived in Arabic countries or traveled outside. Data gathering lasted from January 3, 2022, until March 4, 2022. Persons under 18 were excluded from this survey, while people of Arabic heritage were permitted to participate in this study. To acquire the requisite responders, convenience and snowball strategies were used. The collaborators utilized social media platforms such as Facebook, WhatsApp, and Telegram to disseminate this questionnaire and acquire an acceptable sample. All participants were informed of the study objectives, the name of the research team, their right to quit the study, their right to privacy and data protection, and the knowledge that only fully submitted data would be examined.

### Pilot Study

We tested this questionnaire on 35 random public people to prove it was suitable and easy to understand and answer. Then we made some changes to it from the participants’ comments. Then, using 40 respondents, we ran a pilot test to ensure that the questionnaire’s reliability was validated. Cronbach’s alpha values for the regions ranged from 0.712 to 0.861, indicating that the instrument maintained great internal consistency, and we published the questionnaire once the pilot research was completed.

### Sample Size Calculation

The sample size was calculated using Calculator.net, [https://www.calculator.net/sample-size-calculator.html]. We ran a statistical power analysis for sample size calculation with a population percentage of 50%, a margin of error of 0.02, and a confidence level of 99%. The required sample size was 4161, while the last report of the Arabic population in 2020 was 436.08 million, according to the Statista website. A total of 6478 replies were received; however, 184 respondents—all under 18—did not meet the requirements for statistical analysis, while 233 others declined to provide their permission. The final sample size was 6145 responses.

### Measures

This survey contains the following 43 questions in 5 sections:

1. Socio-demographic characteristics (9 questions): Asking about age [(18–29/30–49/ > 50)], gender, the country (UAE, Saudi Arabia, Qatar, Yemen, Jordan, Syria, Lebanon, Palestine, Iraq, Egypt, Morocco, Libya, Tunisia, Algeria, Sudan, Somalia, Kuwait (, financial status, employment status, educational level, Studying or working in the health sector, and if there are any chronic disease.
2. Knowledge of the COVID-19 pandemic: The responses ranged from Correct and Incorrect to Unsure (7 items). In this section, participants are asked if COVID-19 is a viral or bacterial illness. In addition, this part inquires if the COVID-19 symptoms include fever, cough, sore throat, and shortness of breath. Moreover, if COVID-19 affects persons of any age and whether it transmits through breathe droplets.
3. Preventive measures during COVID-19 pandemic: and classified replays as “true,” “false” (10 items). This section asks participants to choose the most effective COVID-19 prevention method (wash hands after touching surfaces, wash hands before and after touching eyes, nose, or mouth, and wash hands in 6 stages for at least 20 s). In addition to this section, inquire if participants believe wearing a mask, practicing proper sneezing and coughing etiquette, and carrying hand sanitizers consistently are COVID-19 preventative techniques.
4. Knowledge of antibiotics use and resistance: ranged replays from firmly Correct, Incorrect to Unsure (10 items. This section asks participants if antibiotics protect against all types of diseases, if antibiotics expedite the healing process after infection, if antibiotic doses may be adjusted individually, and if antibiotic abuse increases the development of drug resistance.
5. Attitude towards new norm during the COVID-19 pandemic: responses ranged from strongly disagree, disagree, neutral, agree, and strongly agree (7 items). This section asks participants about their attitudes on temperature monitoring in public spaces, the provision of hand sanitizers, the need to wear a mask, and home quarantine for international travelers.

The responses were then categorized as “correct,” “incorrect,” and “unsure,” taking into account both fields of expertise. We were awarded one point for accurate answers and zero for wrong or doubtful responses. In the practice domain, yes answers were given one point; in the attitude domain, a point was given for strongly agreed or agreed answers. The following have been the minimum and maximum score ranges for each domain: e variables were COVID-19 (0–7), antibiotics (0–10), practice (0–10), and Attitude (0–7).

### Statistical Analysis

The statistical package for the social sciences (SPSS) for Windows (version 20.0; IBM) was used to analyze the data and statistically significant set at (*P* value < 0.05). The variables were analyzed descriptively. We also described categorical outcomes as frequency and percentages and continuous variables as means and standard deviations. A one-way analysis of variance (ANOVA) was to be performed to see if the KAP scores differed by sociodemographic factors. The data is given as Mean Standard Deviation (95 percent Confidence interval: Lower Band-Upper band). We used Binominal logistic regression to examine the influence of baseline parameters on the chance that Arabic participants are aware of the COVID-19 pandemic, as well as antibiotic usage and resistance, which we split the two scores into two values (0,1) (Knowledge towards COVID19: 0–4 = 0 and 5–7 = 1 and Knowledge towards antibiotics resistance: 0–5 = 0 and 6–10 = 1). A Pearson’s product–moment correlation was performed to examine the association between KAP scores.

### Ethical Consideration

We obtained the IRB from the Ethical Society for Scientific Research in Syria (IRB = 651-8). In addition, ethical approval was taken from all the contributed Arabic countries. Participation was optional, and replies were fully private; participants were given a URL to access the online survey on Google. The survey asked participants on the first page if they agreed to participate in this questionnaire. They switch to the next page containing complete information about the study before answering the questionnaire. The whole questionnaire may take from 5 to 12 min to complete. All the replies were stored in a secure online database.

## Results

### Demographic Baseline Characteristics

The study sample was collected from 17 Arab countries as follows; Egypt, Syria, and Sudan represented about half (48.8%) of the respondents, while 10.6%, 4%, 1.5%, 7.7%, 8%, 3%, 3.0%, 3.8%, 2.6%, 3.2%, 1%, 1%, 1.3%, 15.9% were from Algeria, Al Kuwait, Iraq, Jordon, Lebanon, Libya, Morocco, Palestine, Qatar, Saudi Arabia, Somalia, Tunisia, UAE, and Yemen, respectively. In total, 6478 responses were received, but 184 individuals did not fulfill the criteria for statistical analysis because they were under 18 years old, while 233 respondents refused consent (3.4%). Six thousand one hundred forty-five people were eligible for statistical analysis. The average age of the respondents was 28.05 years (SD = 9.22, range = 18–76); the majority of the respondents were Male (3193, 52.0%) and had tertiary education (1870, 88.3%), with no medical education background (3115, 50.7%), and with chronic medical illness (5511, 89.7%). A description of demographic data is given in Table 1

**Table 1:** Demographic characteristics of the participants

Characteristics	Frequency	Percentage
Nationality		
Algeria	652	10.6%
Alkawit	27	4%
Egypt	787	12.8%
Iraq	95	1.5%
Jordon	475	7.7%
Lebanon	47	8%
Libya	21	3%
Morocco	183	3.0%
Palestine	231	3.8%
Qatar	157	2.6%
Saudi Arabia	199	3.2%
Somalia	7	1%
Sudan	1158	18.8%
Syria	1045	17.0%
Tunisia	4	1%
UAE	79	1.3%
Yemen	978	15.9%
Age (years)		
18–29	4377	71.2%
30–49	1494	24.3%
Above 50	274	4.5%
Gender		
Male	3193	52.0%
Female	2952	48.0%
Education		
Primary or below	220	3.6%
Secondary	1824	29.7%
Tertiary	4101	66.7%
Occupation		
Full time (government)	1683	27.4%
Partial time (private)	558	9.1%
Student	2207	35.9%
Unemployed	896	14.6%
Retiree	99	1.6%
New graduated	702	11.4%
Medical education background		
Yes	3030	49.3%
No	3115	50.7%
Household income		
Bad (Under 50.000 SP*)	535	8.7
Middle (50.000–100.000 SP)	2404	39.1
Good (100.000–300.000 SP)	2511	40.9
High (Above 300.000 SP)	695	11.3
Chronic disease		
Yes	5511	89.7
No	634	10.3

### Knowledge on COVID-19

The respondents were asked seven questions about COVID-19, and the mean score was 5.45 (SD = 1.30, range 0–7). 77.8% of the respondents had correct answers (5.45/7 * 100). The majority of respondents correctly answered six out of seven questions. However, only about a quarter (1545, 24.1%) knew that COVID-19 could be transmitted without apparent symptoms. Eight hundred eighty-one respondents (13.7%) were unsure whether COVID-19 virus strains could mutate over time (Table 2). COVID-19 knowledge scores varied across genders, age groups, ethnicities, educational levels, occupations, medical education, household income, and regions (Tables 2 and 3).

**Table 2:** Descriptive data of knowledge towards COVID19

No Statement	Correct	Incorrect	Unsure
1. The COVID-19 pandemic is of virus origin	5457 (85.2%)	69 (1.1%)	618 (9.6%)
2. The main clinical symptoms of COVID-19 are fever, cough, sore throat and difficulty in breathing	5795 (90.4%)	74 (1.2%)	275 (4.3%)
3. COVID-19 is highly contagious	5439 (84.9%)	218 (3.4%)	487 (7.6%)
4. Elderly, children, people with co-morbidities and immunocompromised personnel develop more complications if infected	5357 (83.6%)	258 (4.0%)	529 (8.3%)
5. COVID-19 virus is spread mainly through respiratory droplets	4712 (73.5%)	237 (3.7%)	1195 (18.6%)
6. Transmission of COVID-19 virus can only happen when a person developed symptoms	1545 (24.1%)	3422 (53.4%)	1177 (18.4%)
7. COVID-19 virus strain can mutate over time	5132 (80.1%)	131 (6.2%)	881 (13.7%)

**Table 3:** Summary of KAP scales (COVID19, antibiotics use, preventive measures and attitude towards new norm)

No. KAP	COVID19	Antibiotics	Practice	Attitude
Mean	5.45	4.31	3,03	5.2
Median	6	4	3	6
Minimum	0	0	0	0
Maximum	7	10	10	7

Knowledge of COVID19 score was statistically significantly different between all demographic characteristics, while it was higher in females (5.49 ± 1.23) than males (5.37 ± 1.28), and it was higher in the persons who have medical education background(health career, doctor, medical student) (5.68 ± 0.98) (Table 4). The binominal logistic regression model was statistically significant, X2(14) = 275, *p* value < 0.001. The model explained 10% (Nagelkerke R Square) of the variance in knowledge on COVID-19. Of the seven predictor variables, only three were statistically significant: education level, medical education background, and household income (economic level) (Table 5). The number of persons with medical education backgrounds was four times higher than others.

**Table 4:** Differences in knowledge on COVID-19, knowledge on antibiotics resistance, practice and attitudes score with demographic characteristics (one way-ANOVA)

Variable	Knowledge on antibiotics resistance		Knowledge on COVID-19		Practice scores		Attitude scores		
	Mean ± SD (95% CI: Lower–Upper)	*p* value	Mean ± SD (95% CI: Lower–Upper)	*p* value	Mean ± SD (95% CI: Lower–Upper)	*p* value	Mean ± SD (95% CI: Lower–Upper)		*p* value
Age, years(Total) 18–29 30–49 Above 50	4.30 ± 2.40(4.24–4.36) 4.31 ± 2.37(4.24–4.38) 4.21 ± 2.45(4.09–4.34) 4.60 ± 2.47(4.30–4.89)	0.043	5.45 ± 1.30(5.41–5.48) 5.47 ± 1.26(5.44–5.51) 5.36 ± 1.41(5.29–5.43) 5.51 ± 1.35(5.35–5.67)	0.095	2.98 ± 0.11(2.98–2.99) 2.98 ± 0.11(2.98–2.99) 2.98 ± 0.11(2.98–2.99) 2.98 ± 0.15(2.96–3.00)	0.615	5.24 ± 1.77(5.20–5.29) 5.30 ± 1.71(5.25–5.35) 5.10 ± 1.89(5.00–5.19) 5.20 ± 2.02(4.96–5.44)		0.097
Gender(Total) Male Female	4.18 ± 2.32(4.10–4.25) 4.13 ± 2.34(4.03–4.24) 4.22 ± 2.31(4.11–4.33)	0.285	5.34 ± 1.26(5.39–5.47) 5.37 ± 1.28(5.31–5.43) 5.49 ± 1.23(5.43–5.55)	0.005	2.92 ± 0.44(2.90–2.93) 2.92 ± 0.44(2.90–2.94) 2.92 ± 0.45(2.90–2.94)	0.876	5.20 ± 1.63(5.15–5.26) 5.22 ± 1.62(5.15–5.30) 5.18 ± 1.64(5.10–5.26)		0.464
Education(Total) Primary or below Secondary Tertiary	4.18 ± 2.32(4.10–4.25) 4.02 ± 2.50(3.57–4.71) 4.14 ± 2.31(3.90–4.15) 4.27 ± 2.32(4.17–4.37)	0.012	5.43 ± 1.26(5.39–5.47) 4.37 ± 1.61(4.56–5.30) 5.45 ± 1.26(5.30–5.44) 5.93 ± 1.24(5.34–5.54)	0.001	2.92 ± 0.44(2.90–2.93) 2.94 ± 0.35(2.86–3.02) 2.90 ± 0.50(2.87–2.93) 2.93 ± 0.40(2.91–2.95)	0.145	5.20 ± 1.63(5.15–5.26) 4.87 ± 1.77(4.46–5.27) 5.27 ± 1.57(5.19–5.36) 5.17 ± 1.66(5.10–5.24)		0.053
Occupation(Total) Full time (government) Partial time (private) Student Unemployed Retiree New graduated	4.18 ± 2.32(4.10–4.25) 4.01 ± 2.34(3.81–4.21) 4.15 ± 2.32(3.83–4.47) 4.09 ± 2 .27(3.99–4.20) 4.71 ± 2.69(4.45–4.96) 5.66 ± 3.61(1.87–9.46) 4.19 ± 2.05(4.00–4.39)	0.002	5.43 ± 1.26(5.39–5.47) 5.40 ± 1.34(5.29–5.52) 5.22 ± 1.56 (5.00–5.44) 5.42 ± 1.19(5.37–5.47) 5.44 ± 1.38(5.31–5.57) 4.66 ± 2.58(1.95–7.37) 5.60 ± 1.10(5.49–5.70)	0.032	2.92 ± 0.44(2.90–2.93) 2.92 ± 0.42(2.89–2.96) 0.95 ± 0.36(2.89–3.00) 2.91 ± 0.47(2.89–2.93) 2.93 ± 0.39(2.90–2.97) 3.00 ± 0.00(3.00–3.00) 2.92 ± 0.44(2.87–2.96)		5.20 ± 1.63(5.15–5.26) 5.15 ± 1.64(5.01–5.28) 4.84 ± 1.81(4.59–5.09) 5.29 ± 1.54(5.22–5.36) 5.15 ± 1.78(4.99–5.32) 5.00 ± 2.75(2.10–7.89) 5.15 ± 1.70(4.98–5.31)		0.021
Medical education background(Total) Yes No	4.18 ± 2.32(4.10–4.25) 4.64 ± 2.04(4.52–4.70) 3.61 ± 2.53(3.52–3.77)	< 0.001	5.43 ± 1.26(5.39–5.47) 5.68 ± 0.98(5.63–5.72) 5.13 ± 1.48(5.06–5.20)	< 0.001	2.92 ± 0.44(2.90–2.93) 2.94 ± 0,50(2.88–2.92) 2.90 ± 0.36(2.92–2.96)	0.014	5.20 ± 1.63(5.15–5.26) 5.39 ± 1.49(5.32–5.46) 4.98 ± 1.76(4.89–5.07)		< 0.001
Household income(Total) Bad (Under 50.000 SP*) Middle (50.000–100.000 SP) Good (100.000–300.000 SP) High(Above 300.000 SP)	4.18 ± 2.32(4.10–4.25) 4.20 ± 2.58(3.87–4.53) 4.33 ± 2.51(4.19–4.47) 4.06 ± 2.23(3.95–4.17) 4.13 ± 1.82(3.95–4.31)	0.030	5.43 ± 1.26(5.39–5.47) 5.18 ± 1.54(4.99–5.38) 5.39 ± 1.36(5.32–5.47) 5.47 ± 1.16(5.41–5.52) 5.55 ± 1.06(5.45–5.65)	0.004	2.92 ± 0.44(2.90–2.93) 2.92 ± 0.43(2.87–2.98) 2.94 ± 0.37(2.92–2.96) 2.91 ± 0.46(2.88–2.93) 2.89 ± 0.55(2.84–2.94)	0.93	5.20 ± 1.63(5.15–5.26) 5.14 ± 1.77(4.91–5.36) 5.36 ± 1.67(5.27–5.45) 5.24 ± 1.53(5.16–5.32) 4.62 ± 1.67(4.45–4.78)		< 0.001
Chronic disease(Total) No Yes	4.18 ± 2.32(4.10–4.25) 4.16 ± 2.32(4.08–4.24) 4.46 ± 2.32(4.12–4.80)	0.093	5.43 ± 1.26(5.39–5.47) 5.50 ± 1.07(5.34–5.66) 5.43 ± 1.27(5.38–5.47)	0.369	2.92 ± 0.44(2.90–2.93) 2.92 ± 0.44(2.90–2.93) 2.94 ± 0.38(2.89–3.00)	0.358	5.20 ± 1.63(5.15–5.26) 5.21 ± 1.62(5.15–5.26) 5.17 ± 1.72(4.92–5.43)		0.816

**Table 5:** Binary logistic regression between the scales that assesse knowledge towards COVID-19 and antibiotics resistance, and demographic characteristics

	Knowledge on COVID-19				Knowledge on antibiotics resistance			
Variable	OR	95%CI for B		*P* value	OR	95%CI for B		*P* value
		Lower	Upper			Lower	Upper	
Age (years) 18–29(Ref) 30–49 Above 50	1.051.36	0.82 0.82	1.35 2.23	0.64 0.22	1.03 1.19	0.85 0.81	1.25 1.73	0.71 0.36
Gender(Male:Ref)	1.12	0.93	1.35	0.21	0.94	0.82	1.08	0.41
Education Primary or below(Ref) Secondary Tertiary	2.28 2.52	1.54 1.74	3.36 3.66	0.000 0.000	1.09 0.96	0.74 0.66	1.59 1.38	0.64 0.83
Occupation Full time (government) (Ref) Full time (private) Student Unemployed Retiree New graduated	0.92 1.10 1.22 0.73 1.60	0.66 0.82 0.91 0.36 1.04	1.28 1.48 1.64 1.46 2.45	0.65 0.49 0.16 0.38 0.03	1.12 1.47 1.37 0.70 1.12	0.87 1.17 1.09 0.41 0.86	1.45 1.84 1.72 1.20 1.46	0.35 0.001 0.007 0.20 0.36
Medical education Background(No: Ref)	4.03	3.20	5.08	0.000	2.66	2.28	3.10	0.000
Household income Bad (Under 50.000 SP*) (Ref) Middle (50.000–100.000 SP) Good (100.000–300.000 SP) High(Above 300.000 SP)	1.32 1.67 3.23	0.99 1.24 2.06	1.77 2.27 5.05	0.454 0.001 0.000	1.18 1.18 0.26	0.91 0.91 0.19	1.52 1.54 0.34	0.210 0.198 0.000
Chronic disease(No: Ref)	1.14	0.83	1.57	0.403	1.06	0.84	1.35	0.588

The increasing economic level was associated with an increased likelihood of being aware of COVID19. Respondents with high household income (vs. good, moderate, and low household income, OR 3.231, CI 2.066–5.055, *p* = 0.000) obtained higher knowledge scores. However, participants with high household income have a higher probability of good knowledge about COVID-19 than participants with bad household income (OR = 3.23, *P* value < 0.05).

### Knowledge of Antibiotics Use and Resistance

The overall mean of the knowledge of antibiotics resistance was extremely poor (4.31). Respondents were requested to answer ten questions regarding antibiotics’ use and antimicrobial resistance. The mean score was 4.30 (SD = *2.40, range 0–10) among the respondents, resulting in an overall proportion of correct answers of 43% (4.3/10 × 100). In the survey, most respondents got a score of 8 or below, indicating a lack of knowledge about antibiotic resistance, and the overall mean was 4.3 from 10 (Table 6). Eight hundred seventy-one respondents (13.6%) believed that antibiotics could speed the recovery process of all infections. Over half of the respondents (4140, 64.6%) were unaware or uncertain that antibiotics work only against bacterial infections. About half respondents (3014, 47%) did not know or were unsure whether antibiotic resistance would cause mortality (Table 6). There were significant differences in knowledge scores on antibiotic resistance across all demographic characteristics except gender and chronic disease status (Table 4). Furthermore, the binary logistic regression model was statistically significant, X2(15) = 531, *p* value < 0.001. The model explained 13.6% (Nagelkerke R Square) of the variance in knowledge towards antibiotics resistances and usage. Of the seven predictor variables, only the following three were statistically significant: education level, medical education background, and household income (economic level) (Table 5). The persons with medical education backgrounds were 2.6 times higher than others. Student participants and participants with medical education have shown a greater likelihood of good knowledge about antibiotic resistance than respondents with a full-time government occupation and with those with no medical education (OR = 1.47, *P* value < 0.05), (OR = 2.66, *P* value < 0.001), respectively.

**Table 6:** Descriptive data of knowledge on antibiotics use and resistance

No. Statement	Correct	Incorrect	Unsure
1. Bacteria strains can mutate rapidly over time	3292 (51.4%)	805 (12.6%)	2047 (31.9%)
2. Development of new antimicrobials/vaccinations is simple and does not take up much time	988 (15.4%)	3341 (52.1%)	1815 (28.3%)
3. Taking antibiotic can prevent all infection	2020 (31.5%)	2159 (33.7%)	1965 (30.7%)
4. Taking antibiotic can speed up the recovery process of all infection	3524 (55.0%)	871 (13.6%)	1749 (27.3%)
5. Antibiotic dosage dose adjustment can be done without consultation from the professional medical practitioners	1169 (18.2%)	3546 (55.3%)	1429 (22.3%)
6. Antibiotics is effective against bacterial infection only	2004 (31.3%)	1545 (24.1%)	2595 (40.5%)
7. Antibiotic resistance can cause mortality	3130 (48.8%)	565 (8.8%)	2449 (38.2%)
8. Like COVID-19, resistant bacteria strain can cause similar pandemic events	2891 (45.1%)	629 (9.8%)	2624 (40.9%)
9. Misuse of antibiotics will accelerate the antibiotic resistance process	4291 (67.0%)	438 (6.8%)	1415 (22.1%)
10. Hand hygiene is essential to prevent antibiotic resistance	2871 (44.8%)	1488 (23.2%)	1785 (27.9%)

### Practice of Preventive Measures

The practice scores of preventive measures during COVID-19 were measured using ten questions. The mean score was 2.92 (SD = 0.44, range 0–10), giving a 29.2% reduction in well-being practices (2.29/10 × 100). During the COVID-19 pandemic, most (*x*, *z*%) of the respondents practiced at least nine preventive measures. The least practiced preventive measure was avoiding chatting and speaking at close distance (687, 11.1%) (Table 7). In addition, the overall mean of this scale was the lowest scale in our study (3.03).

**Table 7:** Descriptive data of practice of preventive measures during the coronavirus disease 2019 (COVID-19) pandemic

No. Statement	Yes	No
1. Frequent hand washing after in contact with frequent touched surfaces	2792 (45.5%)	3353 (54.5%)
2. Wash hand before and after touching eyes, nose and mouth	1736 (28.2)	4409 (71.7%)
3. Wash hand for at least 20 s	1577 (25.6%)	4568 (74.3%)
4. Wear face mask in public area	2346 (38.1%)	3799 (61.8%)
5. Close mouth and nose when sneezing or coughing	2723 (44.3%)	3422 (55.6%)
6. Always bring along sanitizer or wet wipes	1082 (17.6%)	5063 (82.3%)
7. Always maintain physical distancing at least 1 m from others	1091 (17.7%)	5054 (82.2%)
8. Avoid crowded and narrow places	2768 (45.0%)	3377 (54.9%)
9. Avoid chatting and speaking at close distance	687 (11.1%)	5458 (88.8%)
10. Limit physical contact: no handshake policy, greeting with hand on the chest	1575 (25.6%)	4570 (74.3%)

Respondents were asked seven questions to determine their attitudes toward the new COVID-19 guidelines. The mean score was 5.20 (SD = *1.63, range 0–7), which indicates an overall 74.2% positive attitude in the population (5/2/7 × 100). One-third of respondents strongly agreed that quarantine should be mandatory for all foreign arrivals (2027, 33.0%). However, over half strongly agreed that face masks should be mandatory in all public areas (3229, 52.5%). About two-thirds (644, 59.3%) of the respondents strongly agreed or agreed that working from home is productive (Table 8), and the average mean of this scale was moderate (5.2).

**Table 8:** Descriptive data of attitude towards new norm during the COVID-19 pandemic

No. Statement	Strongly disagree	Disagree	Neutral	Agree	Strongly agree
1. Body temperature monitoring should be practiced at all public areas	195 (3.2%)	763 (12.4%)	1432 (23.3%)	1652 (26.9%)	2174 (35.4%)
2. Availability of hand sanitizer in public area will encourage frequent hand cleaning	48 (0.8%)	136 (2.2%)	369 (6.0%)	2029 (33.0%)	3563 (58.0%)
3. Face mask wearing should be made mandatory in all public area	108 (1.8%)	339 (5.5%)	813 (13.2%)	1656 (26.9%)	3229 (52.5%)
4. Work from home is productive and should be encouraged	193 (3.1%)	813 (13.2%)	1495 (24.3%)	1704 (27.7%)	1940 (31.6%)
5. Table distancing at restaurant should be continued	102 (1.7%)	479 (7.8%)	917 (14.9%)	2336 (38.0%)	2311 (37.6%)
6. Quarantine should be made mandatory for all arrival from overseas	407 (6.6%)	763 (12.4%)	1296 (21.1%)	1652 (26.9%)	2027 (33.0%)
7. Continuous education from the government had helped me to face this pandemic better	59 (1.0%)	78 (1.3%)	338 (5.5%)	1648 (26.8%)	4022 (65.5%)

### Correlations Between Different Domains

There was a perfect positive correlation between the knowledge scores on COVID-19 and the knowledge scores on antibiotics (*r* = 1, *p* = 0.001), and a weak but significant correlation between the practice scores (*r* = 0.36, *p* = 0.001) and attitudes scores (*r* = 0.117, *p* = 0.001). Correlation between attitude and practice scores was also significant but weak (*r* = 0.16, *p* * 0.001) (Table 9).

**Table 9:** the matrix correlation between the utilized assessment tools

Correlations			Knowledge on Covid-19 Scores	Knowledge on Antibiotics Scores	Practice Scores	Attitude Scores
Knowledge on COVID-19 scores			1	–	–	–
Knowledge on antibiotics scores	0.36 (*P* value < 0.001*)	1	–	–		
Practice scores	0.018 (*P* value: 0.135)	0.03 (*P* value: 0.01)	1	–		
Attitude scores	0.16 (*P* value < 0.001*)	0.34 (*P* value < 0.001*)	0.027 (*P* value: 0.036*)	1		

## Discussion

In an effort to evaluate the effectiveness of public health education systems, several KAP studies on COVID-19 were carried out in different parts of the world to evaluate the effectiveness of public health education systems. To address the real knowledge gap in the public and to create more effective teaching techniques, it is crucial to monitor the evolving COVID-19 situation regularly. This is the first large-scale study in the Middle East to investigate the KAP of antibiotic use and COVID awareness. Our study revealed that the general Arabic population has a fair amount of information about COVID-19 and a modest level of acceptability for the new standard. There was little awareness of antibiotic usage, resistance, and prophylactic procedures. According to our results, the mean score for knowledge of COVID-19 was 5.45, and 77.8% of the respondents knew COVID19. These results are similar to a Saudi Arabia study [12], where the total knowledge test accuracy was 81.64%, and the mean COVID-19 knowledge score was 17.96. A National Survey Study in North-Central Nigeria showed more knowledge (99.5%) [13] 83.6% of participants correctly responded that the elderly, children, people with co-morbidities, and immunocompromised personnel develop more complications if infected by COVID-19. This percentage is consistent with another study conducted in Malaysia, which revealed that 89.6% of respondents said not all persons with COVID-19 will develop into severe cases. Only those who are elderly and have chronic illnesses are more likely to be severe cases [14]. Only 24.1% of respondents were aware that COVID-19 might spread without any obvious symptoms. In contrast, 76.2% of the participants in the Nigerian study believed that it is possible to be infected without showing any symptoms [13]. It is easily noticeable that the public of Arab countries possesses relatively low knowledge and awareness levels compared to parallel surveys in other countries. The main reason for such low consciousness towards COVID-19 is the unprepared governmental plans to face similar epidemics. The overall bad socioeconomic status could also be impacting public knowledge. We believe good knowledge promotes preventive actions, and understanding such correlation is vital for any wanted adjustment. The poor knowledge of antibiotics usage and resistance is neither new nor surprising, as most Arab countries have faulty drug restriction policies. It is widely known that antibiotic access is more liberated and facilitated in Arab countries. Thus, misuse problems and inaccurate opinions towards antibiotics will inevitably be found in such an enabling environment.

Other intriguing findings regarding the Practice of Preventive Measures were that just 28.2% of participants replied yes when asked if they washed their hands before and after touching their eyes, nose, and mouth. Curiously, other Chinese results found that 99.7% of respondents regularly washed their hands, and 94% avoided touching their faces with filthy hands [15]. A survey done in Cameron revealed identical findings, with 94.5% cleaning their hands [16]. 38.1% of participants in this poll stated they would wear a face mask in public, and 88.8% said they would not avoid chatting and speaking at close range. Comparing these findings with China and Cameron’s study’s findings, we noticed that 100% of China’s Poll used face masks while engaging in outdoor activities and 95.7% avoided going outside, and 95.8% maintained a 3-m space from everyone. Similarly, 83.8% of participants in Cameron indicated practicing social distancing or did not go to crowded places [15, 16].

Only a third of respondents are with continuing table distancing in restaurants. This is drastically lower than the 84.9% of respondents in the Malaysian survey who believe likewise [9]. The government plays a major role in educating people about the continuous risk of COVID-19 despite the presence of the new vaccines. About two-thirds of respondents have faced COVID-19 more efficiently due to the government’s constant education. This indicates that Arab governments have proclaimed their role in pandemic education to an acceptable level. Knowledge of Antibiotics Use and Resistance was an engaging topic in our research. When asked whether creating new vaccines and antibiotics was easy and did not need much time, we revealed that 15.4% of people answered yes, while 52.1% said no. 31.5% believed that taking antibiotics can prevent all infections; on the other hand, 33.7% did not believe. Our findings on this topic were superior to those of Malaysian research, which showed that 82.9% of participants replied yes when they asked if developing new antimicrobials/vaccinations is simple and does not take up much time. In comparison, 8.2% gave an incorrect response. On the other hand, 64.7% believed antibiotics could prevent all infections, but only 17.4% did not [9]. About two-thirds of respondents have faced COVID-19 more efficiently due to the government's constant education. This indicates that Arab governments have proclaimed their role in pandemic education to an acceptable level. It is easily noticeable that the public of Arab countries possesses relatively low knowledge and awareness levels compared to parallel surveys in other countries. The main reason for such low consciousness towards COVID-19 is the unprepared governmental plans to face similar epidemics. The overall bad socioeconomic status could also be impacting public knowledge. We believe good knowledge promotes preventive actions, and understanding such correlation is vital for any wanted adjustment. The poor knowledge of antibiotics’ usage and resistance is neither new nor surprising, as most Arab countries have faulty drug restriction policies. It is widely known that antibiotic access is more liberated and facilitated in Arab countries. Thus, misuse problems and inaccurate opinions towards antibiotics will inevitably be found in such an enabling environment.

A new Omicron variant appeared in late 2021 with higher transmissibility and lower severity than previous variants [17]. Understandably, the public point of view towards Omicron consisted of both fear and anxiety concerning the new rises of the high-spreading type. Omicron's true danger was the time it spread when most countries started to drop their restrictions [18]. We have noticed some interesting results in attitude towards the new norm of the COVID-19 survey. About one-third of respondents still encourage working from home and believe in being productive. While in the Malaysian survey [9], almost half of the respondents agreed with the working from home concept. This slight difference may be because most Arab countries do not possess the infrastructure to support working from home, and most employees lack the “working from home” experience.

Seven COVID-19-related questions were asked, and the average score was 5.45 ± 1.30 (range 0–7). The majority of respondents (77.8%) correctly answered six out of seven questions about COVID-19 knowledge, while in prior research by Naser et al., only 66% of respondents had a favorable response [19]. Another study by Yang et al. has demonstrated even a higher score of 85.2% regarding Chinese residents’ knowledge and practice in preventing and controlling COVID‐19 [20]. The participants fared the poorest (24%) on the question of whether COVID-19 could be transmitted without symptoms, comparable to the results of the research by Nasir et al., in which the weakest performance was also associated with disease transmission [19]. The participants with a tertiary level of education had better knowledge (4.27 ± 2.32) of antibiotic resistance and better knowledge of COVID-19 (5.49 ± 1.24) with statistically significant results with *p* value being 0.01 and 0.012, respectively. However, those with a secondary education level had better attitudes and practice scores. On the other hand, those with a high school diploma had superior attitudes and practice scores. As a result, having more education does not imply having better attitudes or actions. This was also supported by Naser et al. finding that those with higher levels of education had higher COVID-19 knowledge scores [19]. In keeping with the above, a similar KAP study by Zhong et al. concluded that better education positively correlated with better scores [21]. Moreover, in our study, higher knowledge scores were obtained by respondents in the 30–49 age group, people with medical education, and people with high household incomes.

Regarding the use of antibiotics, a higher education level was observed with better knowledge of resistance. This finding is consistent with previous studies done in the literature on various samples [22–24]. Respondents were asked to answer ten questions about antibiotic use and antimicrobial resistance. The mean score among respondents was 4.30 ± 2.40 resulting in an overall proportion of correct answers of 43%. Moreover, the majority of respondents in the survey received a score of 8 or lower, indicating a lack of knowledge about antibiotic resistance. Over half of the respondents (4140, 64.6%) were unaware or uncertain that antibiotics work only against bacterial infections. About half of respondents (3014, 47%) did not know or were unsure whether antibiotic resistance would cause mortality. All these results were congruent with a study done in Malaysia by Chang et al., who also reported a decreased awareness of antibiotic use in the general population [9]. Overall, those with a better household income showed better knowledge of antibiotic use, which could be due to easy access to educational resources [9, 25, 26]. Our respondents’ lack of antibiotic awareness implies an increased risk of antibiotic abuse, which may promote worldwide antibiotic resistance [27, 28].

Meanwhile, antibiotics information was seldom conveyed through antibiotics awareness initiatives, which the general people may be unaware of. Because there were no consequences, the general population was not obligated to follow any antibiotic use guidelines. This can be a possible explanation for an increased degree of knowledge of COVID-19 when compared to antibiotic usage. During the COVID-19 pandemic, most responders took at least nine preventative steps. Avoiding conversing and speaking at close distance (687, 11.1%) was the least adopted preventative strategy, while respondents favored hand washing after touching contaminated surfaces the most. Those with a greater educational background were shown to have statistically significant superior preventative measures. Students were found to have a better attitude toward the norms than government or private employees, which was not consistent with another study done in Malaysia that showed better attitudes of civil servants towards the norms [9].

Moreover, those with a medical background and those with a middle-range income were also found to have better attitudes towards the norms. This could be due to a better belief in preventive measures and the knowledge of the benefits of adjusting to the norm.

o It is recommended, therefore, to expand COVID-19 viral awareness programs, the necessity of using a face mask correctly, the importance of vaccinating to avoid severe cases, and the importance of social distance.
o We propose establishing online COVID-19 awareness sessions for school and university students.
o Upon recognizing any signs of COVID-19, it is also suggested that they should immediately isolate themselves, conduct a PCR test, and practice strict hand hygiene.
o Others at risk for severe illness from COVID-19 should also be aware of the need to avoid contact with those who have the virus and seek treatment if they test positive for COVID-19.
o The rising incidence of antibiotics self-medication has shown its negative impact; hence, it is crucial to develop awareness initiatives about the threat of antibiotics self-medication.
o Considerations include informing pharmacists not to sell antibiotics without a doctor’s prescription and instituting public education campaigns about the risks of bacterial resistance.

## Limitation

Despite its affordability and usefulness, a cross-sectional research design cannot establish causality at this time. Additionally, the generalizability of this study was improved by using universal sampling and reaching a response rate of 99%, which is greater than the typical response rate for organizational research surveys. Because surveys were anonymous, there was no way to get in touch with respondents after they had completed the forms to check for any unusual responses. It is crucial to confirm that the results of our research cannot be generalized to those who are older, have less education, or do not have access to the internet, all of whom will be excluded from our study. Several steps were taken to increase the study’s dependability with these limitations. To increase the internal validity of study results, use a validated instrument in addition to controlling for potential confounders in the final model and sample from various research locations. A priori sample size calculations are also performed to ensure the study is powerful.

## Conclusion

The Arabic population’s overall mean knowledge of antibiotic resistance and preventative measures scales was 4.31 and 3.03, respectively. As the fourth wave of this pandemic passed, there was also a modest knowledge of the COVID19 pandemic and attitude toward the new norm of COVID19 (OMICRON). In particular, given that many Arabic nations experience conflicts and deteriorating economic and health conditions, world health agencies should reevaluate this common Arab knowledge in order to prevent future deterioration.

## Data Availability

All data related to this paper's conclusion are available and stored by the authors. All data are available from the corresponding author on a reasonable request.

## Abbreviations

KAP: Knowledge, attitudes and practice
SD: Standard deviation
